# Generation of three-dimensional human neuronal cultures: application to modeling CNS viral infections

**DOI:** 10.1186/s13287-018-0881-6

**Published:** 2018-05-11

**Authors:** Leonardo D’Aiuto, Jennifer Naciri, Nicholas Radio, Sesha Tekur, Dennis Clayton, Gerard Apodaca, Roberto Di Maio, Yun Zhi, Peter Dimitrion, Paolo Piazza, Matthew Demers, Joel Wood, Charleen Chu, Jason Callio, Lora McClain, Robert Yolken, James McNulty, Paul Kinchington, David Bloom, Vishwajit Nimgaonkar

**Affiliations:** 1 Department of Psychiatry, University of Pittsburgh School of Medicine, Western Psychiatric Institute and Clinic, 3811 O’Hara Street, Pittsburgh, PA 15213 USA; 2 Thermo Fisher Scientific, Cellular Imaging and Analysis, 100 Technology Drive, Pittsburgh, PA 15219 USA; 3 Department of Medicine Renal-Electrolyte Division and Department of Cell Biology, University of Pittsburgh School of Medicine, 3550 Terrace Street, Pittsburgh, PA 15261 USA; 4 Pittsburgh Institute for Neurodegenerative Diseases, University of Pittsburgh, 3501 Fifth Avenue, BST3-7035, Pittsburgh, PA 15260 USA; 5 Department of Pharmacology and Pharmaceutical Sciences, School of Medicine, Tsinghua University, 30 Shuangqing Rd, Haidian Qu, Beijing Shi, China; 6 Department of Infectious Diseases and Microbiology, University of Pittsburgh, 130 De Soto Street, Pittsburgh, PA 15261 USA; 7 Division of Neurovirology, Department of Pediatrics, Johns Hopkins University School of Medicine, 600 North Wolfe Street, Blalock 1105, Baltimore, MD 21287 USA; 8 Department of Chemistry and Chemical-Biology, McMaster University, 1280 Main Street West, Hamilton, ON L8S4L8 Canada; 9 Department of Ophthalmology, University of Pittsburgh School of Medicine, Suite 820, Eye & Ear Building, 203 Lothrop Street, Pittsburgh, PA 15213 USA; 10 Department of Molecular Genetics & Microbiology, University of Florida College of Medicine, Gainesville, FL 32611 USA; 11 Department of Human Genetics, Graduate School of Public Health, University of Pittsburgh, 130 De Soto Street, Pittsburgh, PA 15261 USA; 12 Department of Pathology, Division of Neuropathology, University of Pittsburgh School of Medicine, 200 Lothrop Street, Pittsburgh, PA 15213 USA; 13 Magee Women’s Research Institute, 204 Craft Ave, Pittsburgh, PA 15213 USA

**Keywords:** Human induced pluripotent stem cells (hiPSCs), Three-dimensional (3D) neuronal cultures, Antiviral drug screening, Herpes simplex virus type 1 (HSV-1), High content screening, Neurodegeneration, CX7 High-Content Screening (HCS) Platform

## Abstract

**Background:**

A variety of neurological disorders including neurodegenerative diseases and infection by neurotropic viruses can cause structural and functional changes in the central nervous system (CNS), resulting in long-term neurological sequelae. An improved understanding of the pathogenesis of these disorders is important for developing efficacious interventions. Human induced pluripotent stem cells (hiPSCs) offer an extraordinary window for modeling pathogen-CNS interactions, and other cellular interactions, in three-dimensional (3D) neuronal cultures that can recapitulate several aspects of in vivo brain tissue.

**Methods:**

Herein, we describe a prototype of scaffold-free hiPSC-based adherent 3D (A-3D) human neuronal cultures in 96-well plates. To test their suitability for drug screening, A-3D neuronal cultures were infected with herpes simplex virus type 1 (HSV-1) with or without acyclovir.

**Results:**

The half maximal inhibitory concentration (IC50) of acyclovir was 3.14 μM and 3.12 μM determined using flow cytometry and the CX7 High Content Screening platform, respectively.

**Conclusions:**

Our A-3D neuronal cultures provide an unprecedented opportunity for high-content drug screening programs to treat human CNS infections.

**Electronic supplementary material:**

The online version of this article (10.1186/s13287-018-0881-6) contains supplementary material, which is available to authorized users.

## Background

Investigations to understand the pathogenesis of neurological and neuropsychiatric diseases have been hampered by the absence of satisfactory human neuronal models. The experimental approaches to modeling central nervous system (CNS) disorders and infections have changed profoundly thanks to human induced pluripotent stem cell (hiPSC) technologies. For the first time, it is possible to generate and manipulate nearly limitless numbers of hiPSC-derived neuronal lineage cells reprogrammed from specific individuals. Such an approach is essential to push the boundaries of personalized medicine since individuals show differences in susceptibility to common environmental, genetic, or infectious stressors. Thus, hiPSC-based models can be used to investigate the pathogenesis of neurotropic viruses and neuropsychiatric and neurodegenerative diseases at the cellular and molecular levels, modeling individual and cell-type specific differences in susceptibility to a given mutation, injury or pathogen, and as cellular platforms for drug screens.

Conventional drug screens employ two-dimensional (2D) cell cultures to enable simple, fast, and cost-effective workflows, but they do not mimic the three-dimensional (3D) architecture of brain tissue in vivo. In recent comparisons between 2D cell monolayers and cells cultured as 3D cellular aggregates, the latter were better predictors of drug responses in vivo [1, 2]. Indeed, cellular responses in 2D monolayer cultures differ from their in vivo analogs for several reasons: 1) impaired cell-cell communication, essential for cells to sense and respond to the environment; 2) reduced intercellular contacts, necessary to forward signaling information to groups of cells; and 3) lack of appropriate extracellular matrix (ECM)-cell adhesion interactions, which play a pivotal role in regulating many physiological processes [3]. The relatively low predictive value of the 2D culture system could account for a portion of drug failures during clinical trials [4]. Hence, there is an urgent need for the development of preclinical models that can more accurately predict clinical outcomes, saving both time and cost.

The first step in the generation of physiologically relevant cellular models is the addition of the “third dimension” to the classic 2D cultures. This is a “*conditio sine qua non*” dictated by the evidence that physiological responses, cell-to-cell signaling, and gene expression profile of 3D cultures more closely resemble in vivo conditions. Over the last decade, substantial literature has demonstrated the superiority of 3D culture systems over the classic 2D monolayer to provide physiological models and predict drug response in vivo [2, 5]. These 3D culture models allow cell-cell and cell-ECM communication, both necessary for an adequate drug response. The use of hiPSCs to generate 3D culture models could revolutionize drug screening. Over the last few years, 3D neuronal cultures have been generated through hiPSC differentiation in scaffolds made of collagen [6], poly(lactic-co-glycolic acid)/poly(l-lactic acid) [7], collagen and hyaluronan [8], alginate [9], polycaprolactone, chitosan, cellulose acetate, and polyethersulfone nanofibers [10, 11]. Furthermore, 3D neuronal cultures have been established within matrices, such as matrigel [5], or as spherical free-floating spheres known as neurospheres [12, 13]. Notably, cerebral organoids, 3D aggregates that recapitulate multiple layers of brain-like regions, have been grown in matrigel droplets [14, 15]. The ability of stem cells to spontaneously aggregate and organize has been reported and is believed to generate multiple neuronal microenvironments in cerebral organoids [16–18]. Cerebral organoids have become popular for modeling aspects of neurodevelopmental disorders, and have great potential to replace the classical 2D cellular platforms employed for drug screening to increase the predictability of drug response in vivo. Despite rapid progress in the development of 3D neuronal cell platforms, their applicability in moderate- to high-throughput drug screening is hindered by the technical complexity of the differentiation procedure and cost. Hence, the replacement of 2D cultures by 3D cultures in drug screening campaigns will require rapid, robust technologies amenable to analysis in a reliable manner, ideally using standard equipment. In this study, we investigated the feasibility of generating adherent 3D (A-3D) neuronal cultures from hiPSC lines derived from two different individuals. Using herpes simplex virus type 1 (HSV-1) infection as an example, we further demonstrate the utility of A-3D cultures grown in 96-well plates for CNS antiviral drug screening.

## Methods

### Cell lines

hiPSC lines 73–56,010-02 and PPMI-51625 were employed in this study. hiPSC line 73–56,010-02 was generated from fibroblasts derived from skin biopsy samples that were collected from a healthy individual via 4-mm full thickness punch biopsies under local anesthesia. The hiPSCs were established at the National Institute of Mental Health (NIMH) Center for Collaborative Studies of Mental Disorders-funded Rutgers University Cell and DNA Repository (http://www.rucdr.org/mental-health) (RUCDR). PPMI-51625, derived from a female Parkinson’s disease patient expressing *LRRK2-G2019S*, was obtained from the Parkinson’s Progression Markers Initiative (PPMI) (http://www.ppmi-info.org/access-data-specimens/request-cell-lines/). As such, the investigators within PPMI contributed to the design and implementation of PPMI and the collection of patient cells for reprogramming to hiPSCs, but did not participate in the analysis or writing of this report. For up-to-date information on PPMI, visit www.ppmiinfo.org.

The samples were labeled without any identifying information. The subjects who provided these samples signed an informed consent that included permission for secondary analyses of samples.

### Generation of A-3D neuronal cultures from iPSCs

hiPSC lines 73–56,010-02 and PPMI-51625 were obtained from RUCDR and PPMI as described above. Monolayer cultures of neural precursor cells (NPCs) were derived from hiPSCs 73–56,010-02 and PPMI-51625 as previously described [19]. 3D neuronal cultures were generated in optical quality (Nalgene Nunc, 400 μl volume, CC2 surface, sterile) 96-well plates treated with matrigel. To induce neuronal differentiation, matrigel was removed and hiPSC-derived NPCs were seeded at a density of 2 × 10^5^ cells/well. NPCs were cultured in neurobasal medium supplemented with 2% B27, BDNF 10 ng/ml, GDNF 20 ng/ml, CHIR9901 3 μM, forskolin 10 μM, dorsomorphin 1μM, 50 U/ml penicillin G, and 50 mg/ml streptomycin. After 4 days, CHIR990, forskolin, and dorsomorphin were withdrawn and cells were cultured for an additional 6 weeks. Half of the culture medium was changed every other day. Cell viability in A-3D cultures was determined by flow cytometry, as described below. Cell viability was also analyzed using propidium iodide (PI) staining; cells were counterstained with Hoechst 33,342 for normalization.

### Generation of 2D neuronal cultures from hiPSCs

NPCs derived from hiPSC lines 73–56,010-02 were seeded in matrigel-coated 96-well plates at a density of 5 × 10^4^ cells/well and cultured in neurobasal medium supplemented with 2% B27 and BDNF 10 ng/ml for 6 weeks. Half of the culture medium was changed every other day.

### Immunocytochemistry

Immunocytochemistry was performed as previously reported [19]. Cells were fixed in 4% paraformaldehyde and permeabilized with 0.2% triton-X. Primary antibodies used were mouse monoclonal anti-β-tubulin III antibody (clone TUJ1, R&D System, 1:50 dilution), mouse monoclonal anti-MAP2 (EMD Millipore, 1:1000 dilution), chicken polyclonal anti-GFAP (Abcam 1:500 dilution), mouse monoclonal anti-VGLUT1 (Abcam; 1:200 dilution), rabbit polyclonal anti-calbindin (Abcam; 1:500 dilution), rabbit polyclonal anti-vimentin (Abcam; 1:1000 dilution), rabbit monoclonal anti-nestin (Abcam; 1:1000 dilution), mouse monoclonal anti-chondroitin sulfate (Abcam; dilution 1:200), rabbit polyclonal anti-CUX2 (Abcam; 1:200 dilution), rat monoclonal anti-CTIP2 (Abcam; 1:200 dilution), and mouse monoclonal anti-Tau (Invitrogen; 1:500 dilution). Alexa Fluor 488 goat anti-rabbit secondary antibody (Life Technologies, 1:200 dilution), Alexa Fluor 488 goat anti-mouse secondary antibody (Life Technologies, 1:200 dilution), Alexa Fluor 594 goat anti-rabbit secondary antibody (Life Technologies, 1:200 dilution), Alexa Fluor 594 goat anti-mouse secondary antibody (Life Technologies, 1:200 dilution), Alexa Fluor 488 goat anti-chicken secondary antibody (Life Technologies, 1:200 dilution), and Alexa Fluor 488 goat anti-rat (Abcam, 1:200 dilution) were used for detection. Counterstaining was performed with TO-PRO-3. A Leica SP5 CW-STED confocal (in normal confocal mode) was used to collect a virtual stack of optical sections taken from the sample. A 40× oil objective was used for acquisition with a magnetic resonance scanner, with sequential scanning for proximal wavelengths, at 0.5 μm optical section thickness, a line average of 8 and frame average of 6, 1024 × 1024 resolution, using conservative laser power settings and HyD detectors. The resultant image z-stacks were compiled into 3D images using LAS AF software, version 4.0 (Leica).

### HSV-1 infections

We used a genetically engineered HSV-1 construct carrying enhanced green fluorescent protein (EGFP) under the control of the viral promoters ICP0, and monomeric red fluorescent protein (RFP) whose expression is driven by the viral promoter glycoprotein C [20]. EGFP expression in infected cells indicates that HSV-1 has entered lytic cycles, while RFP expression indicates commitment to viral DNA replication. Cells were pretreated for 24 h with increasing concentrations of acyclovir (ACV) ranging from 0.1 μM to 50 μM, after which cell-free virus was adsorbed onto cells for 2 h at a multiplicity of infection (MOI) of 0.3. The inocula were removed, cells washed, and then cultured in the presence of ACV. Cells were analyzed at 48 h postinfection (hpi).

### Flow cytometry analysis

At 48 hpi, cells in A-3D and 2D cultures in 96-well plates were washed with phosphate-buffered saline (PBS) and treated with accutase (Sigma) for 15 min and 3 min, respectively, at 37 °C. Single-cell suspensions were generated by pipetting up and down. The cells were transferred into conical 96-well plates and pelleted by spinning down for 15 min at 2500 rpm. The supernatants were discarded, and the cells were washed with PBS, pelleted, and stained with ‘fixable’ viability dye (LIVE/DEAD Fixable Aqua Dead Cell stain kit; Molecular Probes) for 20 min at room temperature, and then washed. The cells were pelleted and resuspended in 2.5% formalin. The percentage of fluorescent cells and cell viability were determined by flow cytometry (FC) using a LSR-Fortessa cell analyzer (Becton, MD).

### High-content imaging

At 48 hpi, cells in A-3D cultures cells were washed with PBS and fixed in 4% paraformaldehyde (PFA) in PBS for 20 min at room temperature. The nuclei were counterstained with Hoescht 33,342. The multiwell plates were loaded into a Thermo Fisher Scientific Cellinsight CX7 high-content imaging platform for automated confocal image capture and analysis. The system is based on an inverted epifluorescence microscope featuring a nipkow-spinning disc confocal that automatically focuses and scans fields in individual wells using a motorized stage. Fluorescent images were produced using a dichroic filter and corresponding emitter filters that pass the fluorescent light onto a high-resolution charge-coupled device camera for image acquisition. Objects were identified as cells if they had valid nuclei and cell body measurements based on size, shape, and fluorescence intensity. Acceptable ranges for these parameters were determined in healthy control wells to ensure that aggregated cells and noncellular particles were excluded from analysis. The acquired images were then analyzed simultaneously by the high-content screening (HCS) Studio software, using the compartmental analysis bioapplication to quantify puncta “spot” detection for each valid cell in the image.

## Results

### Generation of A-3D neuronal cultures

**Fig. 1:**
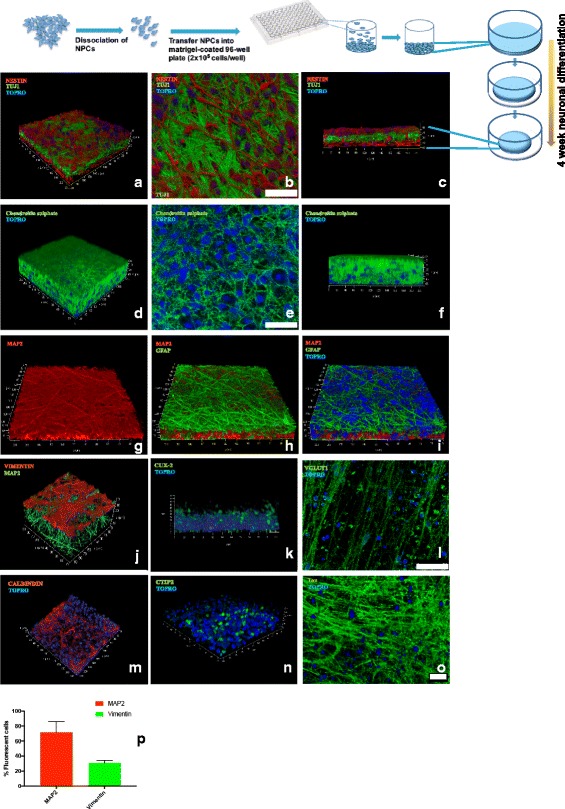
Generation of A-3D neuronal cultures in 96-well plates. The top panel shows a schematic representation of the differentiation procedure. Neural precursor cells (NPCs) are seeded at a density of 2.5 × 10^5^ cells/well on matrigel-treated optical active 96-well plates. Cells are differentiated for at least 4 weeks as described in the Methods section. During this period, NPCs self-assemble and organize into multiple layers. The “spheroidalization” of these multilayered cell aggregates is presumably prevented by the coating of culture wells with matrigel. Matrigel is removed before seeding the cells. During the differentiation process, cells migrate toward the center of the culture wells with a consequent increase in the thickness of the 3D cell aggregates. The images show confocal microscopy analysis of 3D cellular aggregates. Sections (145 μm × 145 μm × 30–55 μm) were generated and are shown in different orientations. **a–c** Staining for Nestin/TUJ1: **a** 3D rendering in an angled orientation; **b** an “en face” view of the 3D rendering; **c** viewing along the depth of the section. **d**–**f** Staining for chondroitin sulfate: **d** 3D rendering in an angled orientation; **e** image of one of the z stacks; **f** viewing along the depth of the section. **g**–**i** Staining for MAP2/GFAP. **j** 3D rendering in an angled orientation of the staining for Vimentin/MAP2. **k** Staining for CUX2 (viewing along the depth of the section). **l** Staining for VGLUT1. **m**,**n** 3D rendering in an angled orientation of the staining for CALBINDIN (**m**) and CTIP2 (**n**). **o** Staining for Tau. **p** Quantification of MAP2-positive and Vimentin-positive cells by high-content imaging. The data represent an average of three independent experiments. Error bars represent standard deviations. Cell nuclei are counterstained with TO-PRO-3 (TOPRO). TOPRO staining shows the presence of multiple cell layers. Scale bar = 20 μm in **b**, **e**, and **o**; = 50 μm in **l**

**Fig. 2:**
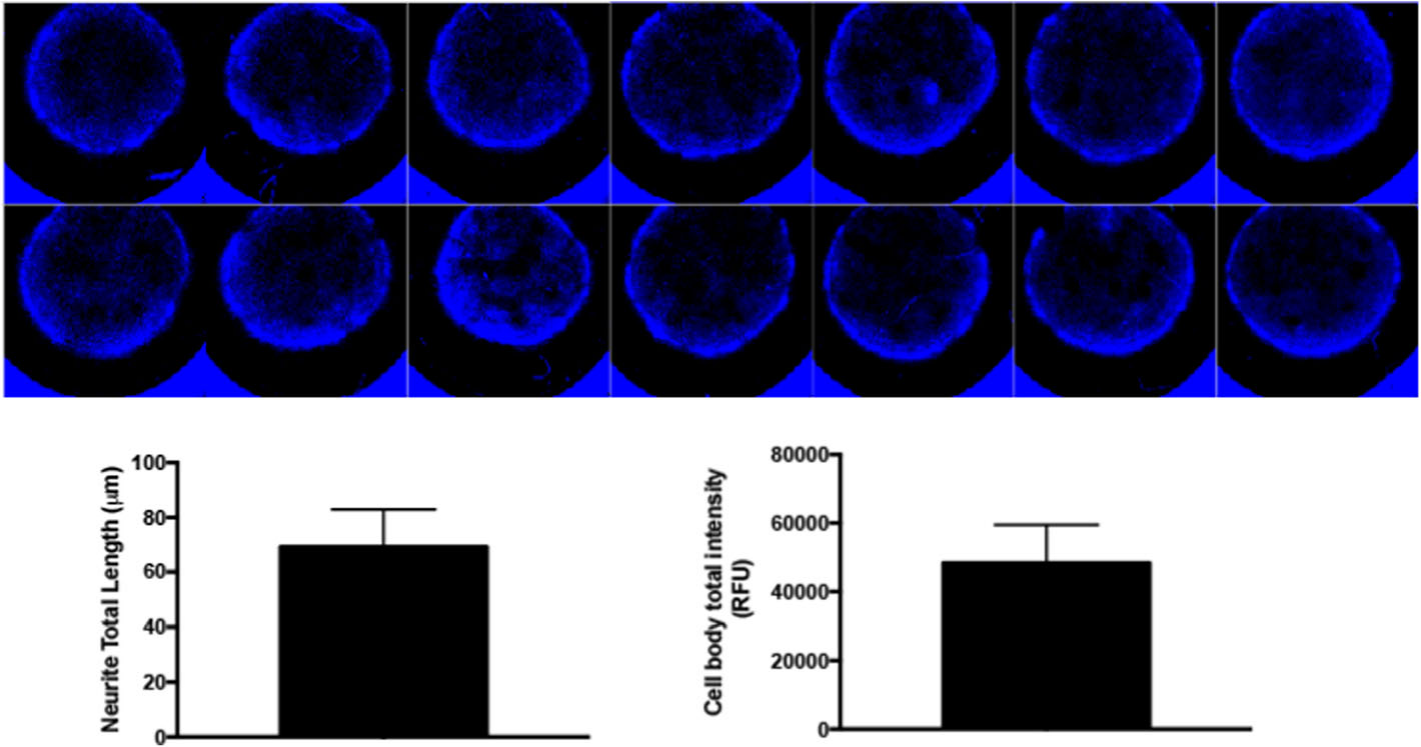
Analysis of well-to-well variability in 3D neuronal cultures. The top image shows Hoechst staining of 3D cultures generated in 96-well plates. The graphs show the average neurite total length (left) and average cell body total intensity (right) and among different culture wells stained with MAP2. The data represent an average of six independent experiments. Error bars represent standard deviations

The strategy for the generation of A-3D neuronal cultures relies on the ability of neural stem cells/neural progenitor cells to self-assemble into aggregates. We refer to the neural stem cells/neural progenitor cells collectively as neural precursor cells (NPCs). NPCs are seeded at high density and, because of their excessive number compared to the available surface in each well, the cells self-assemble in a multilayer fashion. We hypothesized that: 1) the self-assembling and the self-organization of the NPCs and their derived progeny in a 3D space would be accompanied by production of ECM; and 2) the “spheroidalization” of the aggregating cells (leading to the formation of spheres heterogeneous in size) could be prevented by seeding NPCs into matrigel-treated wells. NPCs were generated from hiPSCs line 73–56,010-02 and PPMI-51625 as previously described [19] and seeded on matrigel-treated optically clear 96-well plates at a density of 2.5 × 10^5^ cells/well. The differentiation procedure of NPCs to generate A-3D neuronal cultures is described in the Methods section. During the differentiation process, a higher density of NPCs was observed toward the center of the culture wells, with a consequent increase in the thickness of the 3D cell aggregates (Fig. 1). Our differentiation procedure generated multilayered cell aggregates showing comparable size (Fig. 2), whose thickness ranged from approximately 25 μm to 60 μm after 4 weeks of NPC differentiation (Fig. 1). Furthermore, HCS analysis indicated comparable average cell body total intensity (mean = 69.26 RFU, standard deviation (SD) = 13.66) and average neurite total length (mean = 48,391 μm, SD = 11,111.62) among different culture wells (Fig. 2). After 4 weeks of NPC differentiation, the composition and the distribution of cells in the A-3D cultures was investigated. Cell viability, estimated by flow cytometry using a ‘fixable’ viability dye or by exclusion of propidium iodide, was 80 to 90%.

**Fig. 3:**
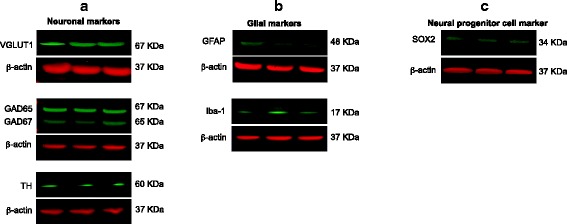
Western blot analysis of the A-3D cultures. **a** Expression of neuronal markers including VGLUT1 (glutamatergic neurons), GAD65/GAD67 (GABA-ergic neurons), and TH (dopaminergic neurons). **b** Expression of glial markers GFAP and Iba-1. **c** Expression of progenitor marker SOX2. Each lane represents an independent biological replicate

Confocal immunofluorescence microscopy of A-3D cultures differentiated from a healthy donor [21] (73–56,010-02) showed a multilayered structure in which neuronal cells expressing the neuronal marker TUJ1 were sandwiched between cell layers expressing the NPC marker nestin (Fig. 1a–c). The production of ECM from differentiating cells in our A-3D cultures was evaluated by analyzing the expression of proteoglycans, the main component of the ECM in the CNS [22]. Figure 1d–f shows abundant staining with chondroitin sulfate proteoglycans (types A and C) which play an important role in the neuronal migration [22], indicating that the ECM produced by the differentiating cells sustains the 3D architecture. The presence of structures resembling developing human cortex was next investigated. A large fraction of cells in the layer above the dense region of MAP2-positive neuronal cells showed glial fibrillary acidic protein (GFAP)-positive (Fig. 1g–i) and vimentin-positive extensions (Fig. 1j) [23, 24] resembling radial glial cells, which are located in the ventricular zone [24]. The mean fraction of cells staining for MAP2 and vimentin were 72.4% (SD = 12.51) and 30.6% (SD 3.37), respectively (Fig. 1p). Staining with the progenitor cell marker CUX-2 (a homeodomain transcription factor expressed in the subgranular zone (SGZ)) was mainly observed in the NPC layer of our 3D cultures (Fig. 1k). CUX2 has several regulatory functions, including the proliferation of the NPCs in the SGZ that will generate upper layers neurons, dendritic growth, and the integration of the upper cortical layers II–III neurons [25]. Western blot analysis indicated that NPCs differentiated into glutamatergic, GABA-ergic, and dopaminergic neurons (Fig. 3). Furthermore, besides the expression of GFAP, Western blot analysis revealed the expression of the microglial marker ionized calcium binding adaptor molecule 1 (Iba1) (Fig. 3). A subpopulation of neurons stained for calbindin (Fig. 1m), a cytosolic calcium-binding protein with greater expression in layer III of pyramidal cells in the dorsolateral prefrontal cortex (DLPFC) compared with layer V pyramidal cells in the same region [26]. Immunoreactivity for CTIP2, a zinc finger protein expressed in layer V neurons, was detected in a subset of neurons [27] (Fig. 1n). Tau protein, which progressively segregates into the axons during neuronal differentiation [28], was abundantly expressed (Fig. 1o). The peripheral area between the 3D cell structure and the edge of the culture well was mainly occupied by processes extending from differentiating cells. In summary, these A-3D cultures showed features of a developing cortex.

To further demonstrate the utility of this method to study CNS disease pathogenesis, we employed this differentiation procedure to generate A-3D cultures using PPMI-51625, a hiPSC line derived from a Parkinson’s disease patient carrying the G2019S mutation in the *LRRK2* gene. This line also spontaneously formed a multilayered organoid structure in which neuronal cells are sandwiched between GFAP-expressing glial cells (Additional file 1: Figure S1). Successful generation of A-3D cultures from both healthy individuals and patients with disease-causing mutations underscores the potential uses of this approach to study cellular-, molecular-, and tissue-based mechanisms and define new neuroprotective therapies for a wide range of neurological disorders.

### Determination of the IC50 for ACV using A-3D cultures

**Fig. 4:**
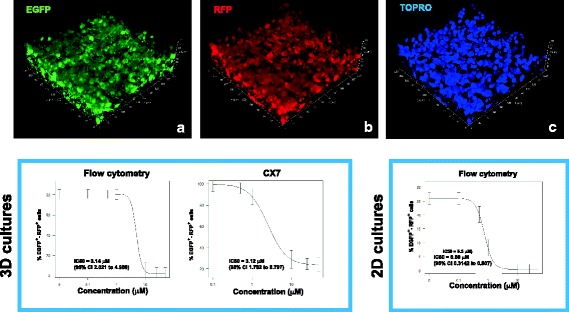
Antiviral efficacy against HSV-1 lytic infection in A-3D neuronal cultures as determined by flow cytometry and CX7 HCS technology. We utilized a genetically engineered HSV-1 construct, incorporating enhanced green fluorescent protein (EGFP) and red fluorescent protein (RFP) as reporter genes, whose expression is driven by the viral promoters ICP0 and glycoprotein C, respectively. The images show confocal microscopy analysis of HSV-1-infected A-3D neuronal cultures depicting the EGFP (**a**) and RFP (**b**) reporter genes. Cell nuclei are counterstained with TO-PRO-3 (TOPRO), (**c**). The graphs show the determination of IC50 for acyclovir in HSV-1-infected A-3D neuronal culture by flow cytometry and CX7 HCS technology (left), and determination of IC50 for acyclovir in HSV-1-infected 2D neuronal cultures by flow cytometry (right). The data represent an average of three independent experiments. Error bars represent model-based standard error

We utilized the neurotropic virus HSV-1 to demonstrate suitability of the A-3D culture system for high-throughput drug screening. A robust, accurate, and rapid analysis of multilayer cellular structures is central to successful employment of 3D cellular platforms for drug screening. We have previously developed 2D models of lytic and latent HSV-1 infections in hiPSC-derived neuronal cells [20, 21, 29]. Our 3D culture models also exhibit lytic infection with HSV-1 (Fig. 4). We therefore tested the antilytic effects of a range of concentrations (0.1 μM to 50 μM) of ACV against a recombinant HSV-1 strain based on KOS virus expressing EGFP and RFP under the control of the viral promoters ICP0 and glycoprotein C (gC), respectively [20], using A-3D cultures of neurons generated from the hiPSC line 73–56,010-02. Cells were pretreated with ACV for 24 h, after which they were infected at a MOI of 0.3 in the presence of increasing concentrations of ACV as described above. Two sets of experiments were performed. Cells were harvested at 48 hpi. In one set of experiments, the number of cells expressing EGFP and RFP was determined by flow cytometry (FC) on a Fortessa FACS analyzer (Becton Dickinson) (see Methods section). In the second set of experiments, the CX7 High-Content Screening (HCS) Platform (Thermo Fisher Scientific) was employed to determine the percentage of fluorescent cells in uninfected and infected wells (see Methods section). The drug concentration that reduced the number of fluorescent cells in cultures infected with HSV-1 by 50% (IC50) was estimated using the four-parameter log*-*logistic function model in the *drc* (V3.0–1) package in R (3.4.0). The IC50 determined by FC and HCS was 3.144 μM and 3.121 μM, respectively (Fig. 4). The similarity between these values derived from traditional FC and high-throughput screening indicates that robust, accurate, and rapid analysis of complex brain cell structures can be achieved using the A-3D culture system.

### Determination of the IC50 for ACV using 2D cultures

To investigate whether ACV exhibits differential efficacy between 2D and 3D cultures, hiPSC-derived monolayer cultures of neurons were generated in 96-well plates (see Methods section). Cells were pretreated with ACV at different concentrations ranging from 0.1 μM to 50 μM and infected with the previously described HSV-1 construct at MOI 0.3. The percentage of fluorescent cells was analyzed using FC. The IC50 of ACV in our 2D neuronal cultures determined using FC was 0.5 μM (Fig. 4).

## Discussion

We have developed a reproducible procedure to differentiate hiPSCs into 3D neuronal cultures in a 96-well plate format that can be used to model CNS infections or genetic contributions to neurological diseases. This A-3D culture system enabled us to model HSV-1 infection and test the antiviral potency of ACV, the gold standard therapy for HSV-1 infections. We showed that the generation of A-3D neuronal cultures does not require that cells be embedded in matrices or seeded into artificial scaffolds. Instead, after an initial period of NPC self-assembly, their differentiating progeny organize themselves in multilayered cell structures showing features of the ventricular/subventricular zone and neuronal cells with some features of human cortical layers. The abundant secretion of ECM, highlighted by immunostaining with chondroitin sulfate (Fig. 1d–f), provides another important advantage with our A-3D platform, considering the influence of ECM on drug activity [3]. We provide evidence for acceptable well-to well variability in our 3D cultures (Fig. 2). We then used two sensitive and quantitative platforms, flow cytometry (FC) and high-content screening (HCS), to determine the IC50 of acyclovir in HSV-1-infected A-3D cultures. The HCS platform yielded estimates comparable to FC (3.1 μM, Fig. 4). The IC50 of ACV estimated in 2D neuronal cultures using FC was 0.5 μM, indicating higher potency of ACV in 2D cell cultures when compared with 3D cell cultures. This may be due to known activities of ECM in binding and regulating the bioavailability of soluble mediators such as drugs and trophic factors, or to a decreased penetrance of ACV in the A-3D cultures.

## Conclusions

Our A-3D human neuronal cultures, paired with a novel confocal-based HCS platform (CX7), provide an unprecedented opportunity to develop a rapid and robust drug screening tool for CNS diseases. There are two distinct advantages of the CX7 HCS technology over FC to analyze 3D cultures. The first comes from the accurate and rapid acquisition of confocal z-stacks from multilayer cellular structures, eliminating laborious and time-consuming manual steps for cell dissociation and staining required for FC. The second advantage is rapid data analysis. To our knowledge, this represents the first evidence for the feasibility of antiviral drug screening in 3D hiPSC-derived neuronal platforms. Our work provides several avenues for future development. Besides additional characterization of A-3D models of CNS infections or neurodegeneration, the scalability of our platform needs to be investigated for optimal drug screening.

## Additional file

Additional file 1: **Figure S1.** Confocal microscopy analysis of A-3D neuronal cultures in 96-well plates generated from the hiPSC line PPMI-51625. (PDF 3272 kb)

## Abbreviations

2D: Two-dimensional
3D: Three-dimensional
A-3D: Adherent three-dimensional
ACV: Acyclovir
CNS: Central nervous system
DLPFC: Dorsolateral prefrontal cortex
ECM: Extracellular matrix
EGFP: Enhanced green fluorescent protein
FC: Flow cytometry
gC: Glycoprotein
HCS: High-content screening
hiPSC: Human induced pluripotent stem cells
HSV-1: Herpes Simplex Virus, type 1
MOI: Multiplicity of infection
NPCs: Neural precursor cells
RFP: Red fluorescent protein
SGZ: Sub-granular zone
