# Supplementary material for: Generation of three-dimensional human neuronal cultures: application to modeling CNS viral infections
Source: Stem Cell Res Ther. 2018 May 11;9:134. doi: 10.1186/s13287-018-0881-6 (PMC5948884; doi:10.1186/s13287-018-0881-6)
Supplement: Supplementary file 1 — Figure S1. Confocal microscopy analysis of A-3D neuronal cultures in 96-well plates generated from the hiPSC line PPMI-51625. (PDF 3272 kb) [file 13287_2018_881_MOESM1_ESM.pdf]

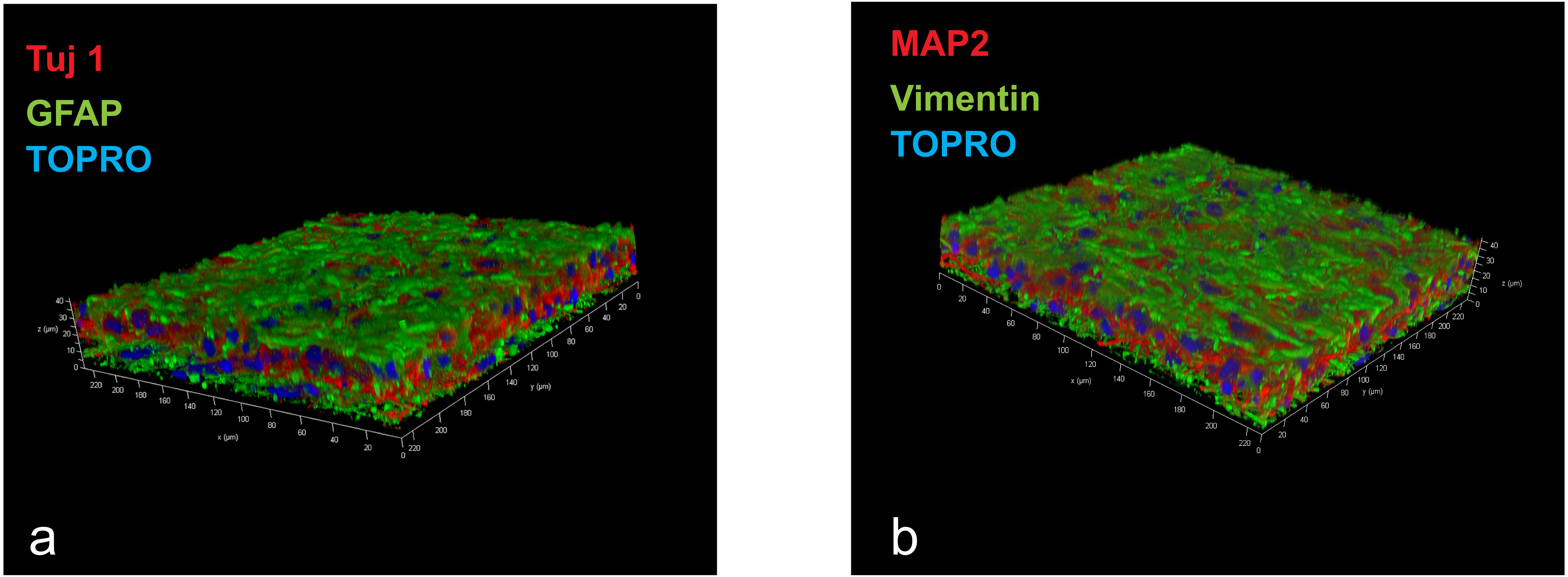

Figure S1. Confocal microscopy analysis of A-3 neuronal cultures in 96-well plates generated from hiPSC line PPMI-51625.
